# Prevalence of syphilis, gonorrhoea and chlamydia in women in Fiji, the Federated States of Micronesia, Papua New Guinea and Samoa, 1995–2017: Spectrum-STI  model estimates

**DOI:** 10.5365/wpsar.2019.10.2.003

**Published:** 2020-03-31

**Authors:** Takeshi Nishijima, Devina Nand, Nefertti David, Mathias Bauri, Robert Carney, Khin Cho Win Htin, Ye Yu Shwe, Anup Gurung, Guy Mahiane, Naoko Ishikawa, Melanie M Taylor, Eline L Korenromp

**Affiliations:** a WHO Regional Office for the Western Pacific, Manila, Philippines.; b Ministry of Health and Medical Services, Fiji.; c Department of Health and Social Affairs, the Federated States of Micronesia.; d National Department of Health, Papua New Guinea.; e Ministry of Health, Samoa.; f UNAIDS, Regional Support Team, Asia and the Pacific, Bangkok, Thailand.; g WHO Country Office, Papua New Guinea.; h Avenir Health, Geneva, Switzerland (ELK), and Glastonbury, USA (GM).; i United States Centers for Disease Control and Prevention, Atlanta, USA.; j Department of Reproductive Health and Research, WHO, Geneva, Switzerland.

## Abstract

**Objective:**

To estimate prevalence levels of and time trends for active syphilis, gonorrhoea and chlamydia in women aged 15–49 years in four countries in the Pacific (Fiji, the Federated States of Micronesia [FSM], Papua New Guinea [PNG] and Samoa) to inform surveillance and control strategies for sexually transmitted infections (STIs).

**Methods:**

The Spectrum-STI model was fitted to data from prevalence surveys and screenings of adult female populations collected during 1995−2017 and adjusted for diagnostic test performance and to account for undersampled high-risk populations. For chlamydia and gonorrhoea, data were further adjusted for age and differences between urban and rural areas.

**Results:**

Prevalence levels were estimated as a percentage (95% confidence interval). In 2017, active syphilis prevalence was estimated in Fiji at 3.89% (2.82 to 5.06), in FSM at 1.48% (0.93 to 2.16), in PNG at 3.91% (1.67 to 7.24) and in Samoa at 0.16% (0.07 to 0.37). For gonorrhoea, the prevalence in Fiji was 1.63% (0.50 to 3.87); in FSM it was 1.59% (0.49 to 3.58); in PNG it was 11.0% (7.25 to 16.1); and in Samoa it was 1.61% (1.17 to 2.19). The prevalence of chlamydia in Fiji was 24.1% (16.5 to 32.7); in FSM it was 23.9% (18.5 to 30.6); in PNG it was 14.8% (7.39 to 24.7); and in Samoa it was 30.6% (26.8 to 35.0). For each specific disease within each country, the 95% confidence intervals overlapped for 2000 and 2017, although in PNG the 2017 estimates for all three STIs were below the 2000 estimates. These patterns were robust in the sensitivity analyses.

**Discussion:**

This study demonstrated a persistently high prevalence of three major bacterial STIs across four countries in WHO’s Western Pacific Region during nearly two decades. Further strengthening of strategies to control and prevent STIs is warranted.

Pacific island countries suffer a high burden of sexually transmitted infections (STIs), but the prevalence and incidence of STIs in individual countries over time are not well known. (*1*)

Two programmatic priorities of the World Health Organization (WHO) Regional Office for the Western Pacific call for expanded STI surveillance. First, in 2017, Member States in the Region endorsed a strategy for the triple elimination of mother-to-child transmission of HIV, hepatitis B and syphilis. (*2*) Mother-to-child transmission of syphilis can cause congenital syphilis, with serious outcomes including miscarriage, stillbirth, neonatal death and congenital abnormalities. (*3*, *4*) Second, the emergence and transmission of antimicrobial resistance in strains of *Neisseria gonorrhoeae*, with documented transmission of multidrug-resistant strains within the Region, highlight the need to enhance monitoring of gonorrhoea and antimicrobial resistance. (*5*) Untreated gonorrhoea and chlamydia can cause pelvic inflammatory disease, infertility and ectopic pregnancy. (*1*, *6*)

The WHO Regional Office for the Western Pacific supports Member States to strengthen national-level STI surveillance and their estimation of the burden of STIs using the Spectrum-STI tool. Spectrum-STI is a module of the Spectrum suite of health policy planning tools, generating country-level estimates of adult prevalence levels of and trends in active syphilis, gonorrhoea and chlamydia infections. The tool, developed by Avenir Health (Geneva, Switzerland, and Glastonbury, CT, USA) at the request of WHO, is available online (https://www.avenirhealth.org) and at no cost. Since 2016, it has been implemented by 10 countries to generate national estimates of STI burden and inform STI surveillance, policies and programmes. (*7*-*12*)

This paper presents Spectrum-STI estimates for Fiji, the Federated States of Micronesia (FSM), Papua New Guinea (PNG) and Samoa, developed during and after a Spectrum-STI training workshop held in April 2018 for national health and surveillance officials. These countries were selected because their burden of STIs was suspected to be high, and their programmes are interested in obtaining estimates.

This paper documents the process for obtaining, the data compilation for and results of the four countries’ estimates, which produced the first-ever national STI estimates for these countries using a standardized, WHO-recommended methodology. Results are discussed with a view to improving national STI surveillance, developing control strategies, and evaluating progress, priorities and challenges.

## Methods

Prevalence trends for adult active syphilis, gonorrhoea, and chlamydia infections were estimated using data from routine national STI surveillance and population-based surveys. No data or records from individual patients were used. We used only published, population-aggregated data sets identified through literature review and official data reported by governments, all of which were fully anonymized (**Supplementary information file**). Data from samples of fewer than 50 people were excluded. For estimates of all three STIs, uncertainty bounds were calculated by bootstrapping (10 000 replications). (*12*)

### Syphilis: prevalence data

Prevalence data were identified from studies conducted between 1995 and 2017 in general populations aged 15–49 years. Searches were conducted on PubMed; government co-authors also searched national (internal) health science databases. Eligible populations included pregnant women receiving antenatal care (ANC; routine screening or sentinel surveys), women attending family planning clinics, and individuals sampled during household surveys or other community-based studies.

Data were adjusted for diagnostic test performance (sensitivity and specificity of the test) and for the contribution of higher-risk populations not represented or underrepresented in population surveys. (*13*-*15*) No adjustments were made for age or location.

Each adjusted data point was assigned a weight reflecting its national coverage and representativeness. Nationally representative data were weighted 100%, while data from smaller areas or from subsets of surveillance sites were weighted proportionally less (**SI file**). (*15*) For example, data from routine ANC screening that covered 60% of pregnant women were weighted 60%; an ANC-based survey that cluster sampled 4 of 10 country provinces was weighted 40%.

### Prevalence estimation: syphilis

Spectrum-STI version 5.72 β 3 (released 20 August 2018) was used. Spectrum-STI has two options for estimating syphilis prevalence trends: segmented polynomial regression and logistic regression. We used logistic regression for Fiji, FSM and Samoa, where the limited data required taking a conservative approach, and polynomial regression for PNG, where the availability of more data over multiple years allowed for more in-depth analysis. (*11*, *15*)

For years occurring before the first data point, Spectrum-STI extrapolated the estimated time trend back 1 year, then kept the estimate constant at that prevalence level. Results are shown for all countries during 2000–2017. However, country data also included the years 1995 through 1999 to best inform estimates for the year 2000. (*15*)

Estimations pooled all women’s prevalence data, from ANC and non-ANC (pregnant and non-pregnant) women, lacking evidence of systematic prevalence differences between these populations. (*12*, *13*, *16*, *17*)

For PNG, the syphilis estimation considered the high local prevalence of yaws across all 22 provinces, which yields false-positive results on both treponemal and non-treponemal tests. (*18*) To correct for false positives, prevalence estimates were multiplied by a correction coefficient of 0.90, which was based on consultation with experts on PNG and published studies on the prevalence of active yaws in the population. (*19*, *20*)

### Gonorrhoea and chlamydia: prevalence data

Prevalence data were identified from studies conducted between 1995 and 2017 in representative populations aged 15–49 years (**SI file**). Eligible populations included pregnant women attending for ANC, women and men attending family planning clinics or undergoing screening at military recruitment, and women and men tested during household or community surveys.

Diagnostic tests eligible for inclusion were nucleic acid amplification tests and culture performed on urine or genital fluid or swabs. The prevalence from each study was adjusted for the sensitivity and specificity of the diagnostic test, as was done for WHO’s 2012 and 2016 global STI estimates and earlier Spectrum-STI estimates for countries. (*10*, *13*) For studies of exclusively rural or urban sites, the test-adjusted prevalence was converted to national prevalence assuming a rural-to-urban ratio of 0.9013 and country year-specific proportions of the urban population. (*21*)

For chlamydia, prevalence data were additionally adjusted for prevalence decline with age. (*10*) To obtain the prevalence for those aged 15–49 years, prevalences from studies that sampled only younger populations (aged 15–24 years) were multiplied by 0.60, and data points from exclusively older populations (aged ≥ 25 years) were multiplied by 1.39. No age adjustments were made for gonorrhoea, as there is a lack of empirical evidence for this.

Similar to syphilis, prevalence data for gonorrhoea and chlamydia were increased by 10% to account for higher-risk populations not represented or underrepresented in general population surveys. (*13*)

Each data point was assigned a weight to reflect its representativeness. Specifically, studies that were representative of the national population were weighted 100%, and other studies were weighted less, reflecting uncertainties in age adjustment and representativeness (**SI file**).

### Prevalence estimations: gonorrhoea and chlamydia

Spectrum-STI fitted a simple moving average through adjusted weighted prevalence data, (*10*, *12*) since prevalence data were insufficient for logistic or polynomial regression. (*13*, *22*) Moving averages used an annual dilution factor (20%), weighting down the contribution of each data point to the estimation for other years by a fixed proportion for each additional year away from the data collection year. (*12*)

### Sensitivity analyses

Univariate sensitivity analyses examined how varying the data inclusion criteria or weights changed the estimated prevalence in 2000 and 2017 (**SI file**). Data added during the sensitivity analyses were weighted 10%. More general methodological assumptions have been addressed in sensitivity analyses of earlier Spectrum-STI applications. (*7*, *12*, *15*)

### Ethics statement

No ethical review was needed because only publicly available information was used.

## Results

### Data availability

All data points are from the years 1995−2017. For syphilis, 8 data points in Fiji, 6 in FSM, 13 in PNG, and 7 in Samoa were identified, all from pregnant women attending ANC clinics (**SI file**). For gonorrhoea, 1 prevalence point for women in Fiji, 9 in FSM, 6 in PNG and 5 in Samoa were identified. For chlamydia, 1 prevalence point for women in Fiji, 10 in FSM, 6 in PNG and 9 in Samoa were identified.

We did not identify any data on male syphilis, so estimates could not be generated; for gonorrhoea and chlamydia, male prevalence data were insufficient to generate estimates for males in any country (**SI file**); hence, estimates were limited to women.

Estimated prevalences are presented as a percentage (95% confidence interval), unless otherwise noted.

#### Fiji

**Figure 1:**
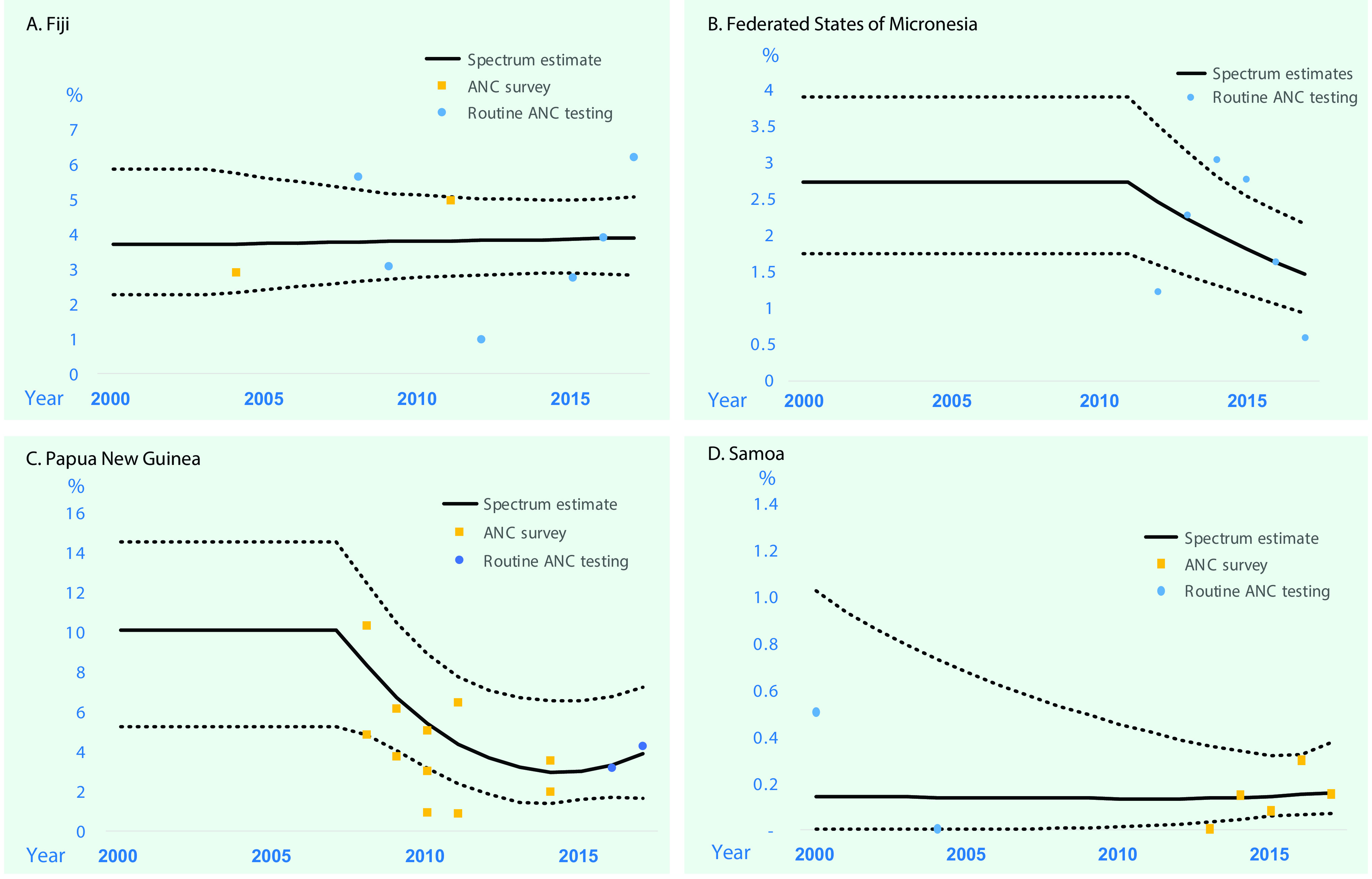
**Estimated trends in prevalence of active syphilis in women aged 15–49 years in Fiji, Federated States of Micronesia, Papua New Guinea and Samoa, 2000 to 2017^a^**

**Figure 2:**
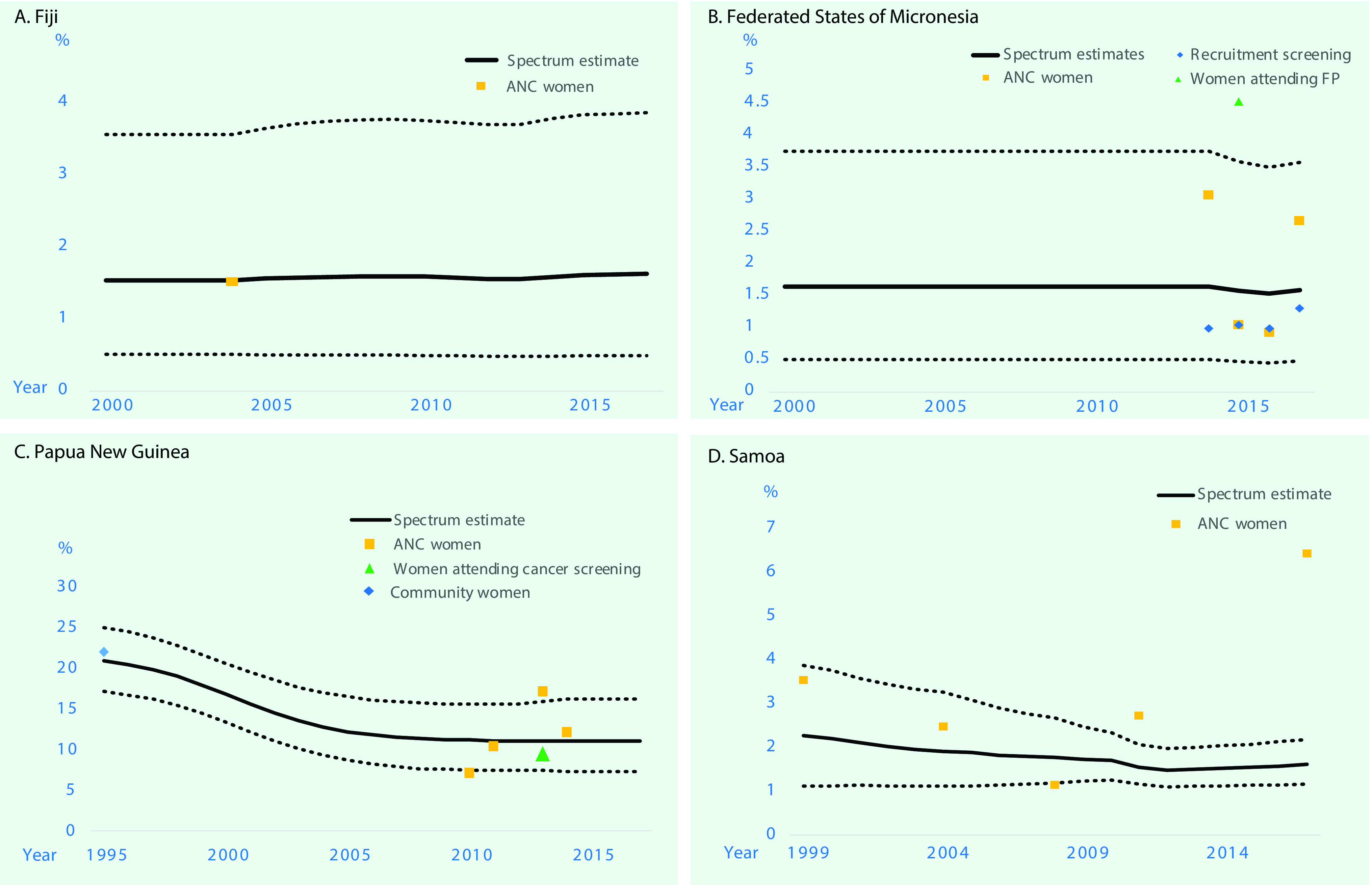
**Estimated trends in prevalence of gonorrhoea in women aged 15–49 years in Fiji, Federated States of Micronesia, Papua New Guinea and Samoa, 2000 to 2017^a^**

**Figure 3:**
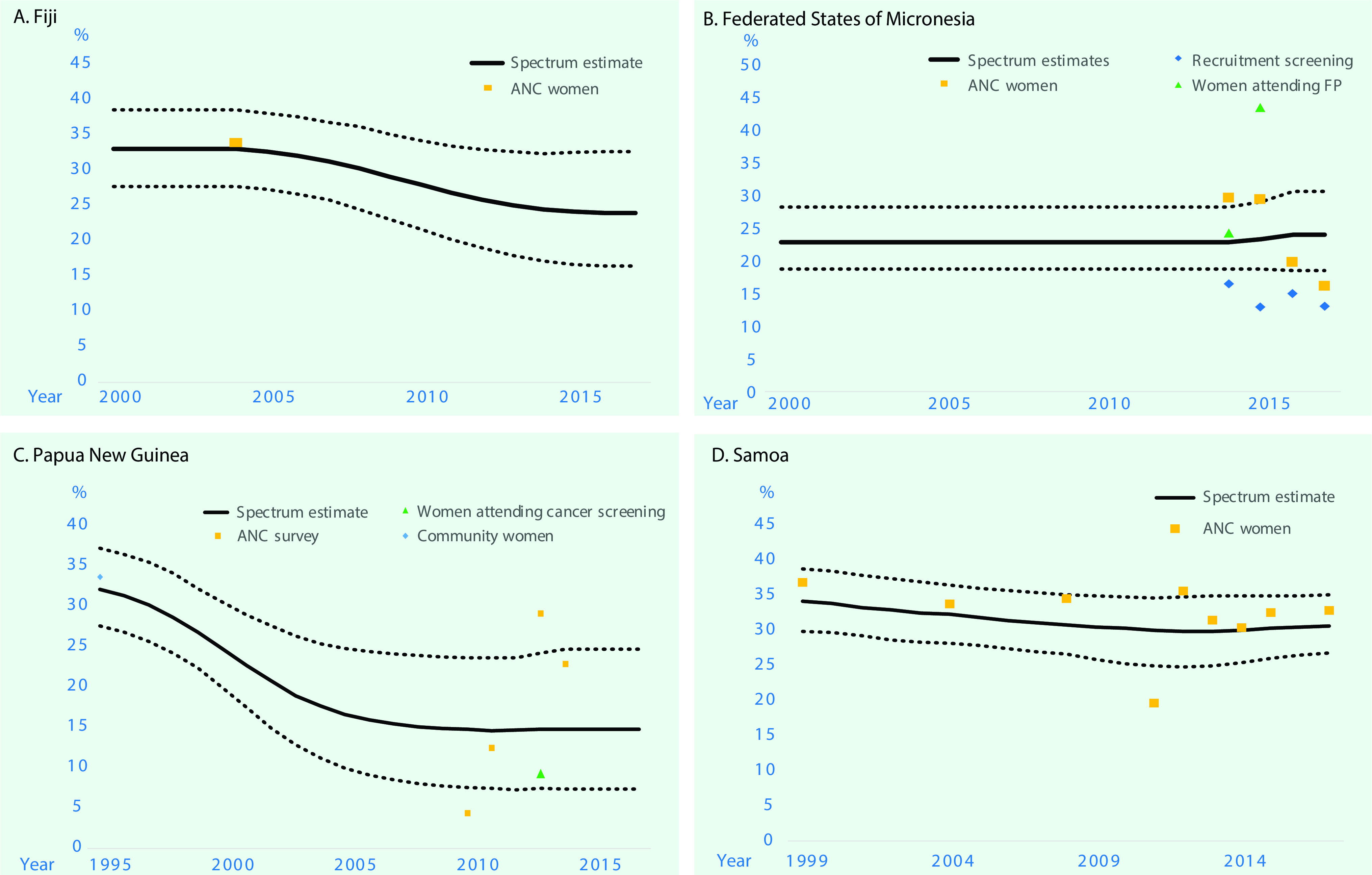
**Estimated trends in prevalence of chlamydia in women aged 15–49 years in Fiji, Federated States of Micronesia, Papua New Guinea and Samoa, 2000 to 2017^a^**

The estimated prevalence of syphilis in 2000 was 3.72% (2.28 to 5.86) and in 2017 was 3.89% (2.82 to 5.06) (**Table 1**; **Fig. 1**). For gonorrhoea, only one prevalence survey was available. We supplemented this with the nine data points from FSM, the country judged most similar in terms of STI epidemiology, underlying drivers and STI-related care. Fiji’s single data point was assigned a weight of 100%, and FSM’s data points were each weighted 10% (**SI file**). The resulting estimates were 1.55% (0.52 to 3.57) in 2000 and 1.63% (0.50 to 3.87) in 2017 (**Table 1**; **Fig. 2**). For chlamydia, the estimation used Fiji’s single data point (weighted 100%), which was supplemented with 10 data points from FSM (each weighted 10%). This gave estimates for 2000 of 33.1% (27.8 to 38.7) and for 2017 of 24.1% (16.5 to 32.7) (**Table 1**, **Fig. 3**).

[**insert** Figure 1]

[**insert** Figure 2]

[**insert** Figure 3]

**Table 1:** Estimated prevalence using Spectrum-STI models of syphilis, gonorrhoea and chlamydia among women aged 15–49 years in Fiji, the Federated States of Micronesia, Papua New Guinea and Samoa, 2000 and 2017

Country	STI	2000^a^		2017^a^	
Fiji	Active syphilis	3.72	(2.28 to 5.86)	3.89	(2.82 to 5.06)
	Gonorrhoea	1.55	(0.52 to 3.57)	1.63	(0.50 to 3.87)
	Chlamydia	33.1	(27.8 to 38.7)	24.1	(16.5 to 32.7)
Federated States of Micronesia	Active syphilis	2.73	(1.76 to 3.91)	1.48	(0.93 to 2.16)
	Gonorrhoea	1.65	(0.51 to 3.75)	1.59	(0.49 to 3.58)
	Chlamydia	22.8	(18.7 to 28.2)	23.9	(18.5 to 30.6)
Papua New Guinea	Active syphilis	10.1	(5.25 to 14.5)	3.91	(1.67 to 7.24)
	Gonorrhoea	16.7	(13.3 to 20.6)	11	(7.25 to 16.1)
	Chlamydia	24.8	(20.0 to 30.6)	14.8	(7.39 to 24.7)
Samoa	Active syphilis	0.14	(< 0.01 to 1.02)	0.16	(0.07 to 0.37)
	Gonorrhoea	2.2	(1.12 to 3.77)	1.61	(1.17 to 2.19)
	Chlamydia	33.8	(29.7 to 38.5)	30.6	(26.8 to 35.0)

^a^ Values are percentage point estimate (95% confidence interval).

Federated States of Micronesia

The estimated prevalences of syphilis in 2000 and 2017 were 2.73% (1.76 to 3.91) and 1.48% (0.93 to 2.16), respectively (**Table 1**; **Fig. 1**). For gonorrhoea, the estimated prevalences in 2000 and 2017 were 1.65% (0.51 to 3.75) and 1.59% (0.49 to 3.58), respectively (**Fig. 2**). For chlamydia, the estimated prevalences in 2000 and 2017 were 22.8% (18.7 to 28.2) and 23.9% (18.5 to 30.6), respectively (**Fig. 3**).

#### Papua New Guinea

The estimated syphilis prevalences in 2000 and 2017 were 10.1% (5.25 to 14.5) and 3.91% (1.67 to 7.24), respectively (**Table 1**; **Fig. 1**). For gonorrhoea, the estimated prevalences in 2000 and 2017 were 16.7% (13.3 to 20.6) and 11.0% (7.25 to 16.1), respectively (**Fig. 2**). For chlamydia, the estimated prevalences in 2000 and 2017 were 24.8% (20.0 to 30.6) and 14.8% (7.39 to 24.7), respectively (**Fig. 3**).

#### Samoa

For syphilis, the estimated prevalences among women in 2000 and 2017 were 0.14% (< 0.01 to 1.02) and 0.16% (0.07 to 0.37), respectively (**Table 1**; **Fig. 1**). For gonorrhoea, the estimated prevalences in 2000 and 2017 were 2.20% (1.12 to 3.77) and 1.61% (1.17 to 2.19) (**Fig. 2**), respectively, and for chlamydia, the prevalences were 33.8% (29.7 to 38.5) and 30.6% (26.8 to 35.0), respectively (**Fig. 3**).

### Differences across countries and sexually transmitted infections

Trend estimates for all three infections in all countries were either stable or suggestive of declines in prevalence, except for syphilis in PNG, which showed a slight increase between 2014 and 2017 after a drastic decline that began in 2007. However, differences between 2000 and 2017 were modest or small for any infection and country, as reflected by overlapping 95% confidence intervals for each specific disease within each country.

Syphilis prevalence in 2017 was higher in PNG and Fiji than in Samoa, but other differences between countries were relatively small, and 95% confidence intervals overlapped between countries.

For gonorrhoea, PNG’s prevalence estimate was above that of the other three countries. For chlamydia, the estimated 2017 prevalence did not differ widely between countries. In all four countries, in 2017, chlamydia was more prevalent than gonorrhoea and active syphilis.

### Sensitivity analyses

For Fiji, alternative estimates for syphilis that increased weights for data from sentinel surveys in 2004 and 2011 (to 100%) produced similar prevalences; only the 2017 point estimate was below the estimate for 2000 in the alternative scenarios, whereas in the default estimate, the prevalence in 2017 was marginally above that for 2000 (**Table 2**). Alternative estimates excluding the data borrowed from FSM yielded estimates similar to those of the default (which included the data from FSM) in terms of levels and trends for both gonorrhoea and chlamydia (**Table 2**). Clearly, time trends in the default estimates were driven by the data from FSM, whereas the single data point from Fiji by definition gave a time-constant estimate.

**Table 2:** Sensitivity analysis showing the effect of varying selected assumptions and values on estimated national prevalence of sexually transmitted infections among women aged 15–49 years in Fiji, the Federated States of Micronesia, Papua New Guinea and Samoa, 2000 and 2017

Country	Syphilis^a^		Gonorrhoea^a^		Chlamydia^a^	
	2000	2017	2000	2017	2000	2017
Fiji						
Default scenario	3.72 (2.28–5.86)	3.89 (2.82–5.06)	1.55 (0.52–3.57)	1.63 (0.50–3.87)	33.1 (27.8–38.7)	24.1 (27.8–38.7)
Excluding 9 data points from the Federated States of Micronesia	-	-	1.42 (0.48–3.25)	1.42 (0.48–3.25)	34 (28.3–39.5)	34 (28.3–39.5)
Increasing to 100% the weight for ANC prevalence surveys (2004 and 2011)	4.12 (1.53–8.40)	3.8 (2.59–5.30)	-	-	-	-

Federated States of Micronesia						
Default scenario	2.73 (1.76–3.91)	1.48 (0.93–2.16)	1.65 (0.51–3.75)	1.59 (0.49–3.58)	22.8 (18.7–28.2)	23.9 (18.5–30.6)
Including 3 data points from surveys with small sample sizes	-	-	2.72 (0.86–6.58)	2.8 (0.85–6.28)	21.6 (17.2–26.7)	20.4 (15.6–25.8)

Papua New Guinea						
Default scenario	10.1 (5.25–14.5)	3.91 (1.67–7.24)	16.7 13.3–20.6)	11.0 (7.25–16.1)	24.8 (20.0–30.6)	14.8 (7.39–24.7)
Adding prevalence data for blood donors, STI clinic patients and female sex workers (9 data points for syphilis; 2 for gonorrhoea and chlamydia)	9.21 (4.65–19.0)	5.68 (2.72–9.13)	16.9 (13.4–21.4)	11.8 (8.03–17.1)	24.7 (19.7–30.2)	15.2 (7.41–24.8)
Logistic regression used for estimates of syphilis prevalence instead of polynomial regression	9.86 (5.05–16.2)	1.43 (0.42–3.50)	-	-	-	-

Samoa						
Default data	0.14 (0–0.94)	0.16 (0.07–0.37)	2.2 (1.12–3.77)	1.61 (1.17–2.19)	33.8 (29.7–38.5)	30.6 (26.8–35.0)
Including data from blood donors and immigration screening (17 data points)	0.2 (< 0.01–1.02)	0.28 (0.09–0.67)	-	-	-	-
Excluding routine gonorrhoea data from 2017 with high prevalence (1 data point)	-	-	2.22 (1.19–3.92)	1.06 (0.81–1.38)	-	-
Including sexually active females sampled in 2015 for chlamydia (1 data point)	-	-	-	-	34 (29.6–38.3)	30.9 (25.9–35.1)
Increasing to 100% the weighting for ANC prevalence surveys (2000 and 2004−2005)	0.17 (< 0.01–1.13)	0.28 (0.10–0.74)	-	-	-	-

ANC: antenatal care.

^a^ Values are percentage point estimate (95% confidence interval).

For FSM, adding three gonorrhoea data points with sample sizes of fewer than 50 people into the analysis, increased the estimated prevalence of gonorrhoea in both 2000 and 2017. For chlamydia, adding two data points from samples with fewer than 50 people had little effect on prevalence estimates for 2000 and 2017, but it did cause the 2017 estimate to be lower than that for 2000. However, for both gonorrhoea and chlamydia, the 95% confidence intervals for each STI overlapped between 2000 and 2017.

For PNG, adding data from the country survey from STI clinic patients, female sex workers (FSWs) (**SI file**) and blood donors (only for syphilis) slightly increased the estimated syphilis prevalence for 2017. Estimates of gonorrhoea and chlamydia remained similar to their default estimates (**Table 2**). For syphilis, substituting polynomial regression for logistic regression did not materially change the estimate for 2000, but it did change the trend: rather than being a U-curve, the prevalence fell so that the 2017 estimate was lower than the 2017 default (**Table 2**).

For Samoa, adding data from blood donors and immigration screening slightly increased the estimated syphilis prevalence. Alternative estimates using weights increased to 100% for sentinel surveys (2000 and 2004−2005) showed prevalences similar to the default − that is, 0.17 (< 0.01 to 1.13) in 2000 and 0.28 (0.10 to 0.74) in 2017 (**Table 2**). For gonorrhoea, excluding data from routine ANC screening from 2017 (which had an outlying high prevalence of 5.63%) slightly reduced the estimated prevalence for 2017. For chlamydia, an estimate that added a 2015 survey on sexually active females (which was excluded from the default because it included only women who did not use condoms for a year, and they were thus considered to be at higher risk) led to a prevalence similar to the default. (*23*)

## Discussion

These first-ever national estimates of prevalence trends among women in four Pacific island countries found persistently high prevalences of syphilis, gonorrhoea and chlamydia. All countries had data from periodic syphilis prevalence screenings collected during routine ANC visits, and in earlier years, there was ANC-based sentinel surveillance. As in past regional and global estimates, (*13*, *17*) the prevalence was highest for chlamydia, which in three countries was followed by gonorrhoea, while active syphilis was less prevalent. An exception was Fiji, where active syphilis was estimated to be more prevalent than gonorrhoea.

For PNG, the country with the most data across the STIs investigated, estimates suggested a recent decline in syphilis, although the 95% confidence intervals for the estimates overlapped for 2000 and 2017. For gonorrhoea and chlamydia, the best estimates suggested a decline in both; however, the 95% confidence intervals overlapped for each disease in 2000 and 2017. The consistency in trends across the STIs adds credibility to these estimates. The apparent decline in syphilis among women may reflect PNG’s 2006 roll-out of syphilis screening for pregnant women as a one-stop ANC-based service using a rapid treponemal test, with those who have positive results being treated with intramuscular benzathine penicillin G on the same day. (*24*) Furthermore, a decline is consistent with PNG’s trend in syphilis prevalence among FSWs identified in integrated biobehavioural surveys from 1995 to 2017. (*25*, *26*)

Nevertheless, this estimated decline in syphilis comes with uncertainty. First, diagnostic algorithms used in ANC testing varied, from rapid plasma reagin (RPR) with *Treponema pallidum* hemagglutination assay (TPHA) confirmation used in surveys during 2008–2014 to routine data from TPHA-based rapid tests used during 2016–2017. Having additional years of routine data should improve certainty regarding a decline in syphilis prevalence.

For gonorrhoea and chlamydia, there is less evidence for declines in prevalence, and they are less plausible. PNG treats gonorrhoea with amoxicillin-based regimens, for which resistance was already detected before 2010 (unpublished data). In response to the high prevalence of gonorrhoea and amoxicillin resistance in antimicrobial susceptibility testing, PNG has revised its recommended first-line treatment for gonorrhoea to combination therapy with cefixime, a broad-spectrum cephalosporin, plus azithromycin, and it started national roll-out of this treatment in 2019. (*27*) A similar treatment guideline revision, recommended by WHO to prevent and control gonococcal resistance, may be indicated for Fiji. Fiji still recommends oral penicillin for treating ulcerative and genital discharge syndromes, which is unlikely to be effective for all gonococcus strains, according to unpublished data on antimicrobial susceptibility from one of its four divisional hospitals (Fiji, Ministry of Health and Medical Services, unpublished data, 2014−2017).

In Samoa, presumptive chlamydia treatment with azithromycin for women attending for ANC and their partners has been in place since June 2015. However, its implementation is hampered by low awareness among providers and target populations, and the lack of an operational strategy for partner tracing. (*28*) This may explain why chlamydia prevalence has not fallen since 2000.

Notably, for PNG, the estimated prevalence levels and trends did not materially change for any STI when data were added from high-risk women attending STI clinics and from FSWs (**Table 2**). (*25*, *29*) Apparently, women in the general population (sampled in ANC and community surveys) are at similarly high risk of STIs as those attending STI clinics and as FSWs. This suggests it would be beneficial to intensify STI prevention and screening services, expanding them beyond syphilis screening during ANC and services targeting FSWs and men who have sex with men (MSM). For example, adding gonorrhoea and chlamydia to ANC screening, as is done in some countries, (*7*) should reduce women’s infection rates and prevent perinatal complications.

### Limitations

The key limitation in these estimates lies in the availability and representativeness of the data, which precluded making firm estimates of trends. Fiji had one single data point each for gonorrhoea and chlamydia; Fiji’s relatively high syphilis estimate (compared with gonorrhoea in Fiji and compared with syphilis in other countries) may be an artefact of limited data. For syphilis, two countries had no data outside that collected during ANC; within the ANC data, two of four countries had at least one change in the diagnostics used during the time for which data were collected, which added possible bias and uncertainty about trends, although we adjusted for test performance.

We identified few prevalence data for men (four surveys on gonorrhoea and chlamydia in general populations in FSM, one survey on syphilis among MSM in PNG, and nothing in the other two countries; **SI file**), and therefore could not make estimates for STIs among men, as were included in earlier Spectrum-STI country-level applications. (*10*) Other estimations have inferred male prevalence from estimates among females, applying a time-constant male-to-female prevalence ratio, for example, for syphilis of 1.0, indicating an equal prevalence among women and men. (*13*, *17*) For PNG, extending the estimated 3.91% prevalence among low-risk women in 2017 to men would appear to be consistent with the 4–8% range observed in a two-site 2017 survey of MSM, (*25*) although the confidence bounds on such a low-risk estimate among males would be wider than the 1.67−7.24% range we estimated for women (**Table 1**).

A special concern for syphilis surveillance in PNG is the endemicity of yaws, which causes positivity on TPHA and RPR tests and false-positive results for syphilis on any diagnostic algorithm. Our estimation adjusted for yaws-attributable false-positive syphilis, but the validity of the adjustment and its constancy over the time horizon that was evaluated (which implies that the prevalence of yaws shares a time trend with syphilis, an assumption we did not explicitly assess) remain uncertain.

Earlier Spectrum-STI estimations triangulated prevalence with national case reports of new incident STIs, to give an indication of the plausibility of estimates, or with clinical treatment coverage and reporting completeness. (*7*, *9*, *10*) We did not pursue such triangulation, as routine clinical STI case reports were not available for FSM and Samoa, and in Fiji and PNG, they were available for only a few years.

### Implications for surveillance and programming

The estimated high prevalence of STIs suggests several areas to be considered to improve STI surveillance and programming.

Controlling gonorrhoea and chlamydia in high-prevalence populations requires offering services beyond clinical treatment and passive clinic-based surveillance, including active outreach, screening of all members of higher-risk populations, strengthening partner testing, and, as shown for PNG, extending these activities beyond known key groups. In women, most gonorrhoea and chlamydia infections are asymptomatic; therefore, etiologic screening, for example, during ANC, may identify many women who would not present to a clinic. Etiologic screening is promoted by WHO, for both control and surveillance purposes. (*30*) Clearly, all countries studied here would benefit from implementing periodic prevalence assessments during ANC and assessments of men and women in the general population. Given these countries’ high STI prevalences, such assessments could be small-scale and yet, with careful site sampling, yield valuable representative data indicative of national time trends. Assessments may benefit from increasing countries’ laboratory capacity, including using new DNA-based platforms, such as the GeneXpert system (Cepheid, Sunnyvale, CA, USA), initially introduced for tuberculosis screening, which are available in PNG, FSM and Samoa for diagnosing gonorrhoea and chlamydia, although capacity is limited as is the geographical reach.

Clinical services should improve their partner notification strategies and links to care, in both primary health-care and specialized STI settings. Although results from programmatic partner notification are not routinely collected, PNG implements partner notification at all health-care settings that provide STI care.

WHO recommends syphilis screening for all pregnant women, preferably during the first trimester. For settings with a syphilis prevalence higher than 5%, WHO recommends one-stop screening during ANC using treponemal-based rapid tests, followed by on-site treatment with benzathine penicillin G to maximize treatment coverage and minimize the loss to follow-up seen during referral for confirmatory testing and treatment by STI specialists. (*31*-*33*) PNG implements this approach. PNG may want to extend one-stop rapid-test-based syphilis screening and treatment to services for FSWs and MSM which would reserve its limited laboratory capacity for performing confirmatory RPR tests. The other three countries still use laboratory non-treponemal and treponemal two-test algorithms even during ANC, so switching to rapid testing would seem beneficial there as well.

This four-country study illustrates the value of using a standardized approach to estimate STI trends. The Spectrum-STI approach provides several advantages over earlier approaches.

It provides an intuitive, free, online tool that national programme managers and surveillance officers can learn to use within a few days during a workshop.
It provides a framework for collating national data with key information elements, notably, diagnostic tests, sample sizes, data coverage, representativeness and quality.
It uses internationally agreed, expert- and evidence-based assumptions about key parameters, such as the duration of infection and sensitivity and specificity of a diagnostic test, to frame and guide estimation, such that estimates are comparable among countries and within countries over time.
Spectrum-STI’s statistical methods have been documented in international, peer-reviewed scientific literature (*12*, *15*, *34*) and accepted for estimating STI trends. (*34*)

For PNG and other countries that also take periodic STI prevalence measurements among FSWs and MSM (**SI file**, for PNG), estimations could be refined to consider these two or other key groups, or some combination of these, independently alongside low-risk women and men. Since late 2018, a new version of Spectrum-STI that allows this type of refinement has been rolled out. (*11*, *35*)

## Conclusions

These first, national-level prevalence estimations for Fiji, FSM, PNG and Samoa confirm persistently high prevalences of the three STIs studied. The data that were available precluded making precise estimates or drawing firm conclusions regarding time trends; however, they underscore a clear need and opportunities for improving STI surveillance, prevention and treatment in these Pacific island countries.
